# Supplementary material for: Distinct roles of XPF-ERCC1 and Rad1-Rad10-Saw1 in replication-coupled and uncoupled inter-strand crosslink repair
Source: Nat Commun. 2018 May 23;9:2025. doi: 10.1038/s41467-018-04327-0 (PMC5966407; doi:10.1038/s41467-018-04327-0)
Supplement: Supplementary file 1 — rad1E706Kmus81Δslx1Δ [file 41467_2018_4327_MOESM1_ESM.pdf]

A

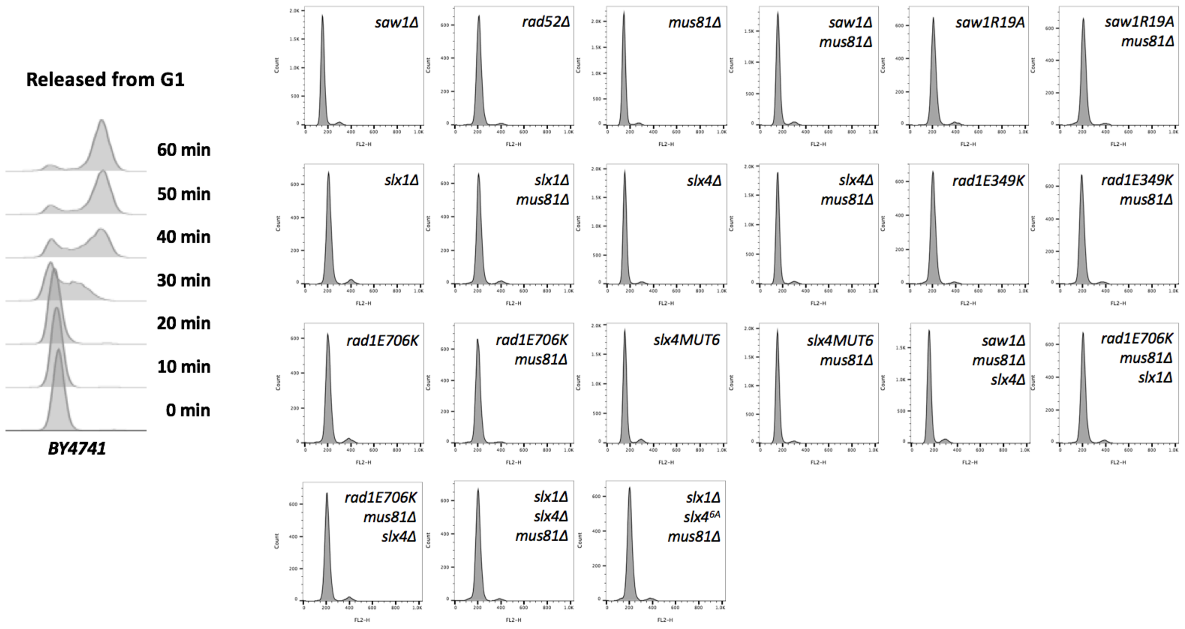

B

α-factor

exponential growth ⟶ G1 arrest ⟶ HN_2 treatment ⟶ YEPD (2h) ⟶ wash ⟶ plating or flow cytometry

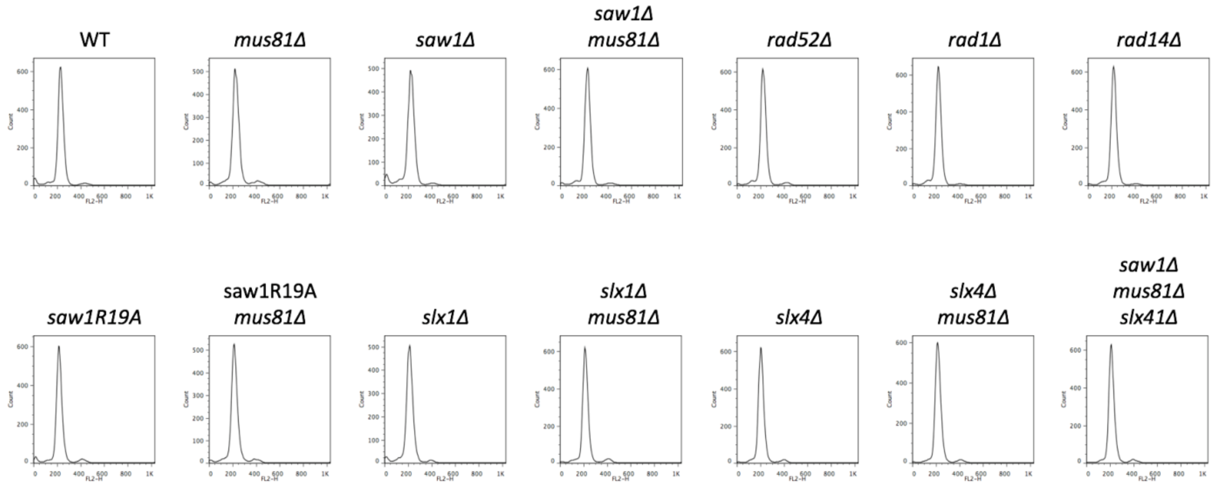

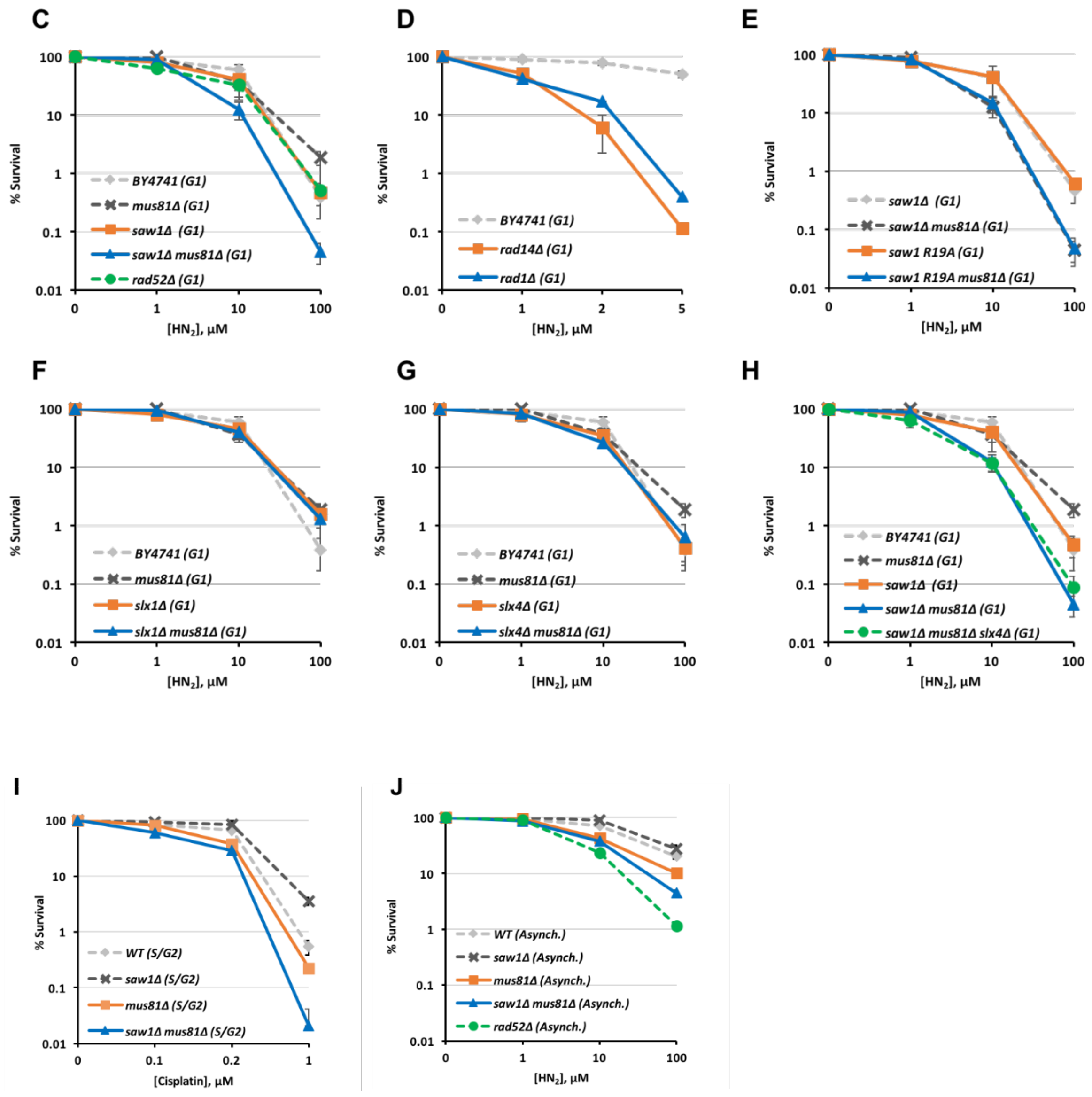
C
% Survival
BY4741 (G1)
mus81Δ (G1)
saw1Δ (G1)
saw1Δ mus81Δ (G1)
rad52Δ (G1)
[HN2], μM
D
% Survival
BY4741 (G1)
rad14Δ (G1)
rad1Δ (G1)
[HN2], μM
E
% Survival
saw1Δ (G1)
saw1Δ mus81Δ (G1)
saw1 R19A (G1)
saw1 R19A mus81Δ (G1)
[HN2], μM
F
% Survival
BY4741 (G1)
mus81Δ (G1)
slx1Δ (G1)
slx1Δ mus81Δ (G1)
[HN2], μM
G
% Survival
BY4741 (G1)
mus81Δ (G1)
slx4Δ (G1)
slx4Δ mus81Δ (G1)
[HN2], μM
H
% Survival
BY4741 (G1)
mus81Δ (G1)
saw1Δ (G1)
saw1Δ mus81Δ (G1)
saw1Δ mus81Δ slx4Δ (G1)
[HN2], μM
I
% Survival
WT (S/G2)
saw1Δ (S/G2)
mus81Δ (S/G2)
saw1Δ mus81Δ (S/G2)
[Cisplatin], μM
J
% Survival
WT (Asynch.)
saw1Δ (Asynch.)
mus81Δ (Asynch.)
saw1Δ mus81Δ (Asynch.)
rad52Δ (Asynch.)
[HN2], μM

Supplementary Figure 1 a Cell cycle profile by flow cytometry. (Left) BY4741 was arrested in G1 phase by alpha-factor for 2 hr and released from G1. Flow cytometry was performed with samples taken by indicated time point. (Right) Indicated gene deletion mutants were arrested G1 phase and confirmed by flow cytometry. **b** Cell cycle profile of cells after arresting in G1 phase and treated with HN_2 for 2 hr. Flow cytometry was performed as described in **a.** **c** BY4741 and indicated gene deletion mutants were arrested in G1 phase (**b-h**) and grown asynchronously (**i**) and treated with various concentrations of HN_2 for 2 hr before plated on YEPD. Shown are the average ± s.d. of three independent experiments.

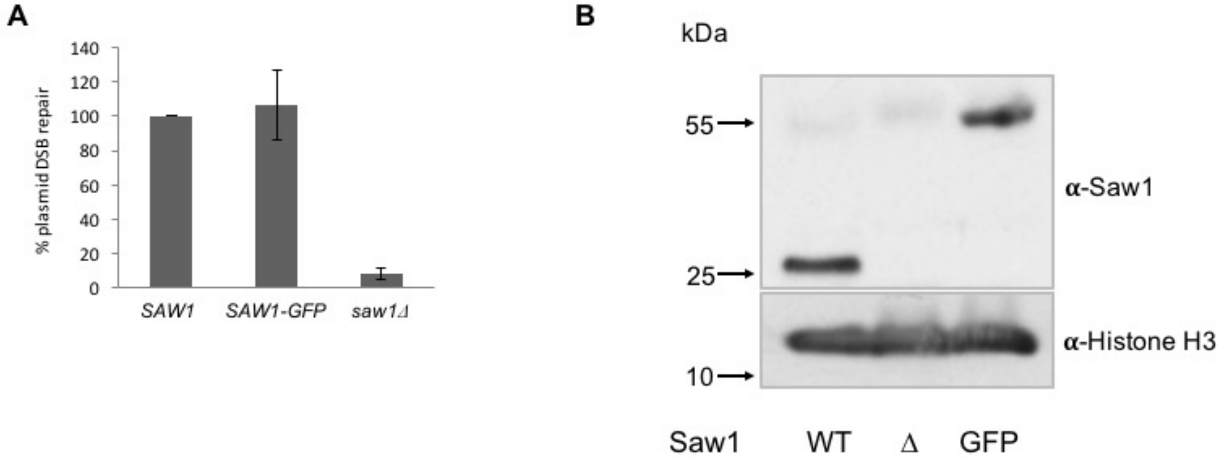

Supplementary Figure 2 a Saw1-GFP is fully capable of promoting SSA between *lacZ* repeats flanking *Bsu*36I site on pNSU208. Percentage of plasmid DSB repair is calculated by dividing the number of transformants with *Bsu36I* digested plasmids with those with undigested plasmids. **b** The intracellular levels of Saw1-GFP and the untagged proteins were visualized and compared in wild type (WT), *saw1*Δ (Δ) and *saw1*Δ/SAW1-GFP (GFP) strains using immunoblot assays with anti-Saw1 antibody. The level of histone H3 was detected using anti-H3 antibody and used as a loading control.

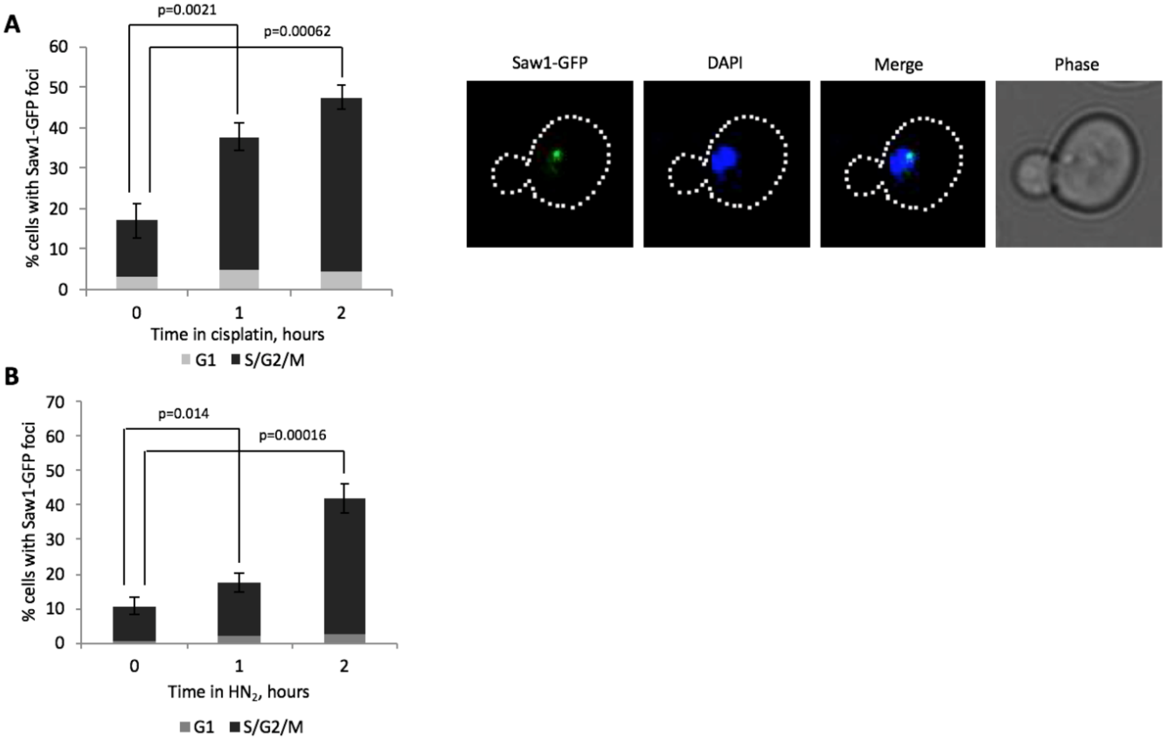

Supplementary Figure 3 Saw1-GFP forms foci in response to CDDP treatment. Representative images of CDDP-induced Saw1-GFP (right) and a graph showing the percentage of cells with Saw1-GFP foci after 2 μM CDDP treatment (**a**) or HN_2 (**b**) at indicated time points (left). Error bars represent standard deviation of three independent experiments with at least 100 cells counted per experiment. Student t-test was used to calculate *P* values on statistical significance of Saw1-GFP foci as compared to those at t = 0.

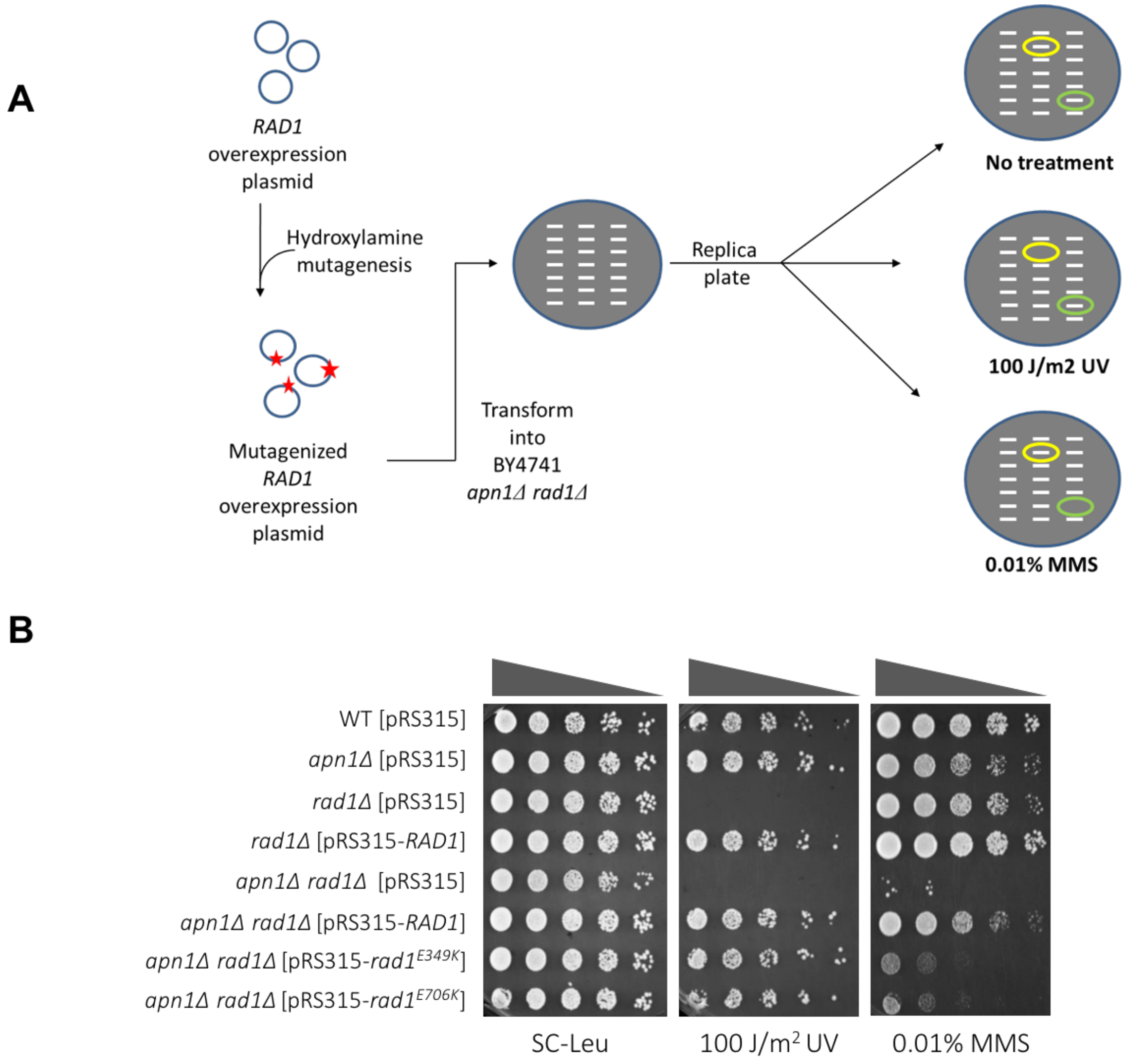

Supplemental Figure 4 Identification of *rad1* mutants deficient in 3' flap removal. **a** Illustration of screening strategy to identify *rad1* mutants deficient in 3' flap removal but proficient in UV lesion repair. **b** Viability of *apn1* gene deleted cells supplemented with plasmids expressing wild type, rad1-E349K or rad1-E706K after 0.01% MMS or 100 J/m_2 UV treatment. Cells were grown to mid-log phase and serially diluted onto medium containing MMS or were irradiated by UV before photographed after 3 days of growth at 30°C.

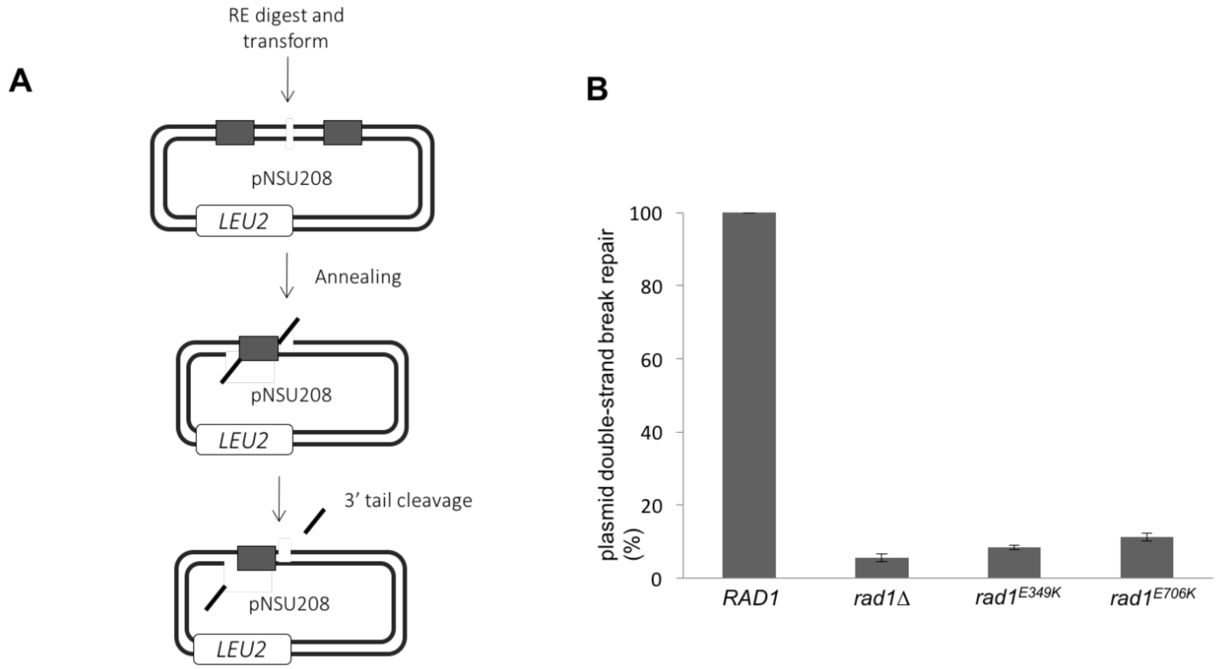

Supplementary Figure 5 The effect of *rad1-E349K* and *rad1-E706K* mutations on plasmid-based SSA. **a** Illustration of plasmid-based SSA reporter (pNSU208). The restriction enzyme (*Bsu*36I) site (RE), *LEU2* marker, and the direct repeat sequence (grey boxes) are shown. **b** Cells expressing rad1 mutations are deficient in repair of linearized pNSU208 by SSA. Indicated strains were grown to mid-log phase and then transformed with *Bsu*36I digested or mock digested pNSU208. Percentage of SSA is calculated by dividing the number of transformants with *Bsu*36I digested plasmids by those with undigested plasmids.

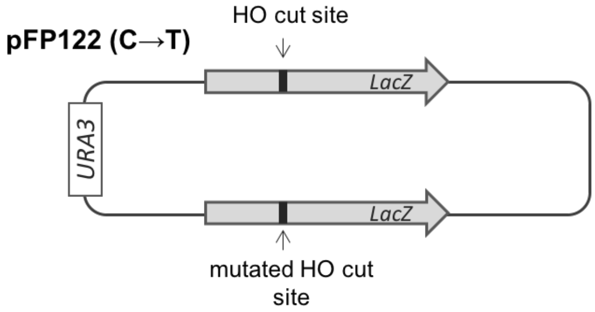

	Plasmid Retention Levels (%)[b]			
Plasmid	WT	*rad1Δ*	*rad1-E349K*	*rad1-E706K*
pFP122 (C→T)[a]	86	75	78	91
pFP121 (10/0)	76	68	72	71
pFP118 (20/0)	62	27	45	36
pFP131 (308/0)	72	16	18	15
pFP130 (0/610)	80	11	16	13
pFP120 (308/610)	56	1.4	1.7	3.4

[a]Numbers in brackets indicate size of 3' non-homologous sequence at left and right sides of the broken ends, respectively
[b]Percentage of plasmid retention was calculated as the ratio of colonies retaining assay plasmids on YEP-galactose media versus those on YEPD. Plasmid retention was calculated from more than 1000 colonies

Supplementary Figure 6 (top) Graphical representation of 3' flap removal reporter plasmids containing a single nucleotide mutation (pFP122, C→T) or 308- and 610-nucleotide deletions (pFP120, non-homology highlighted in blue) at the donor *lacZ* gene, respectively, that form 3' flap during gene conversion upon HO cleavage at the recognition sequence at the recipient *lacZ*. The location of *URA3* marker is shown. (bottom) The results of plasmid retention assay in RAD1+, *rad1Δ*, *rad1-E369K*, and *rad1-E706K* mutants.

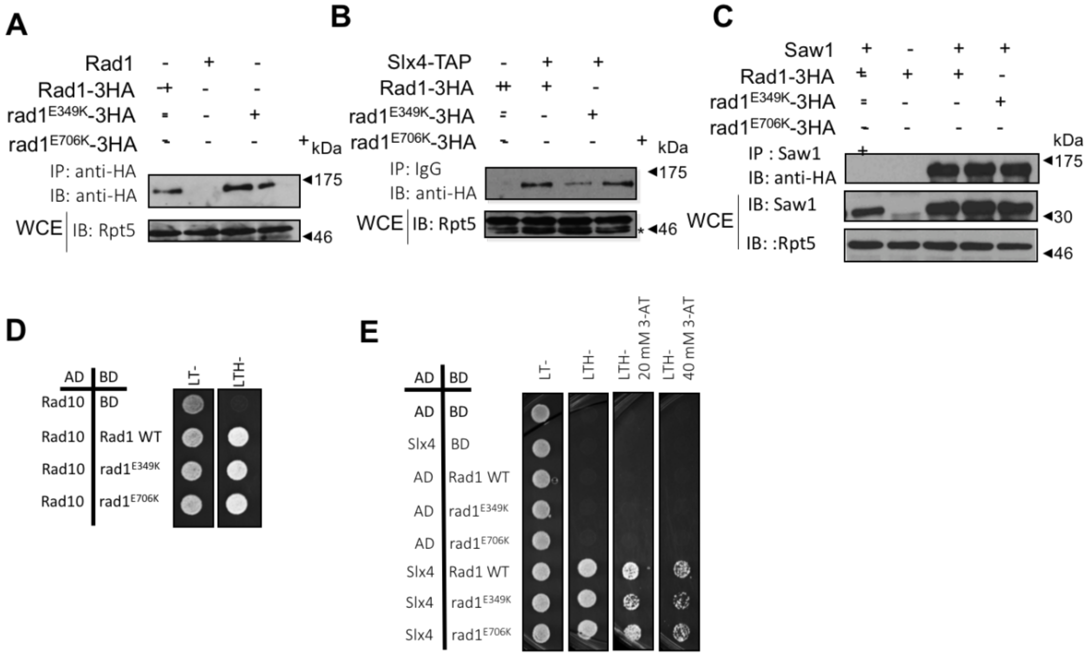
A
Rad1
Rad1-3HA
rad1E349K-3HA
rad1E706K-3HA
kDa
IP: anti-HA
IB: anti-HA
175
WCE
IB: Rpt5
46
B
Slx4-TAP
Rad1-3HA
rad1E349K-3HA
rad1E706K-3HA
kDa
IP: IgG
IB: anti-HA
175
WCE
IB: Rpt5
46
C
Saw1
Rad1-3HA
rad1E349K-3HA
rad1E706K-3HA
kDa
IP : Saw1
IB: anti-HA
175
WCE
IB: Saw1
30
IB: :Rpt5
46
D
AD
BD
LT-
LTH-
Rad10
BD
Rad10
Rad1 WT
Rad10
rad1E349K
Rad10
rad1E706K
E
AD
BD
LT-
LTH-
LTH- 20 mM 3-AT
LTH- 40 mM 3-AT
AD
BD
Slx4
BD
AD
Rad1 WT
AD
rad1E349K
AD
rad1E706K
Slx4
Rad1 WT
Slx4
rad1E349K
Slx4
rad1E706K

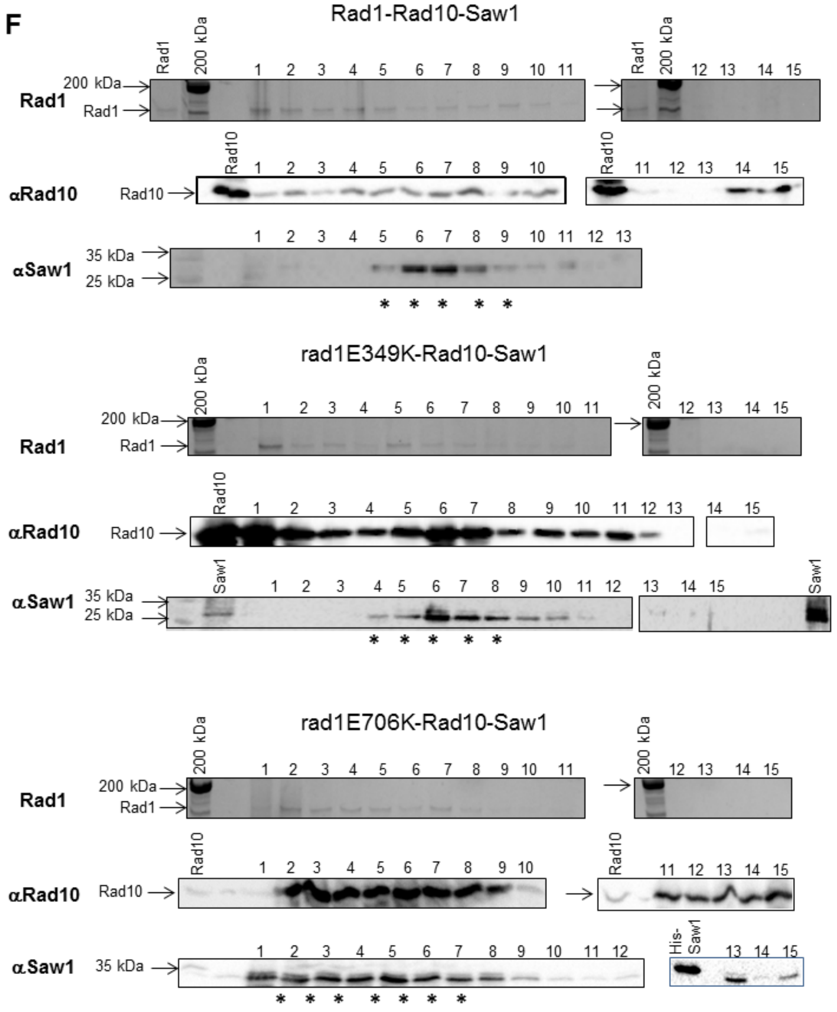
F
Rad1-Rad10-Saw1
Rad1
αRad10
αSaw1
200 kDa
Rad1
Rad10
35 kDa
25 kDa
rad1E349K-Rad10-Saw1
Saw1
rad1E706K-Rad10-Saw1
His-Saw1

Supplementary Figure 7 Integrity of physical interaction between rad1 mutants and other 3' flap removal proteins. **a** The level of expression of rad1-E349K and rad1-E706K proteins. The level of Rpt5 in whole cell lysate was used as a control. **b** Co-immunoprecipitation of Rad1 and Slx4. Slx4-TAP was pulled down by IgG agarose and the level of Rad1 or rad1 mutants were determined by Western blot with anti-HA antibodies. Asterisk indicates non-specific signals in immunoblot with anti-Rpt5 antibodies. **c** Co-immunoprepicitation of Rad1 and Saw1. Saw1 and the associated proteins were immunoprecipitated with anti-Saw1 antibody and the amount of Rad1 and rad1 mutant derivatives were detected by immunoblot with anti-HA antibody. **d-e** Interactions between Rad10 and Rad1 (**d**), and Slx4 and Rad1 (**e**) were determined by yeast two-hybrid assay. Yeast two-hybrid assays were performed with GAL4 DNA binding domain (GBD) fusion of Rad10 or Slx4 and activation domain (GAD) fusion of Rad1 or rad1 mutants to detect interaction between these proteins. (**f**) Analysis of representative His-Rad1/Rad10-Saw1 gel filtration fractions, His-rad1E349K-Rad10-Saw1 and His-rad1E706K-Rad10-Saw1 complexes. Fractions were analyzed for the presence of Rad1 (top; silver stain), Rad10 (middle; Western blot), or Saw1 (bottom; Western blot). Fractions that contain all three proteins are indicated by an asterisk. Purified His-Rad1, Rad10, Saw1 and His-Saw1 were used as size markers where indicated. The 200 kDa molecular weight marker from broad range molecular wight marker (Bio-Rad) is shown on the silver stained gels. The 35 kDa and 25 kDa molecular weight markers from the PageRuler Plus prestained markers (Thermo) were overlaid and shown on the Western blots.

A

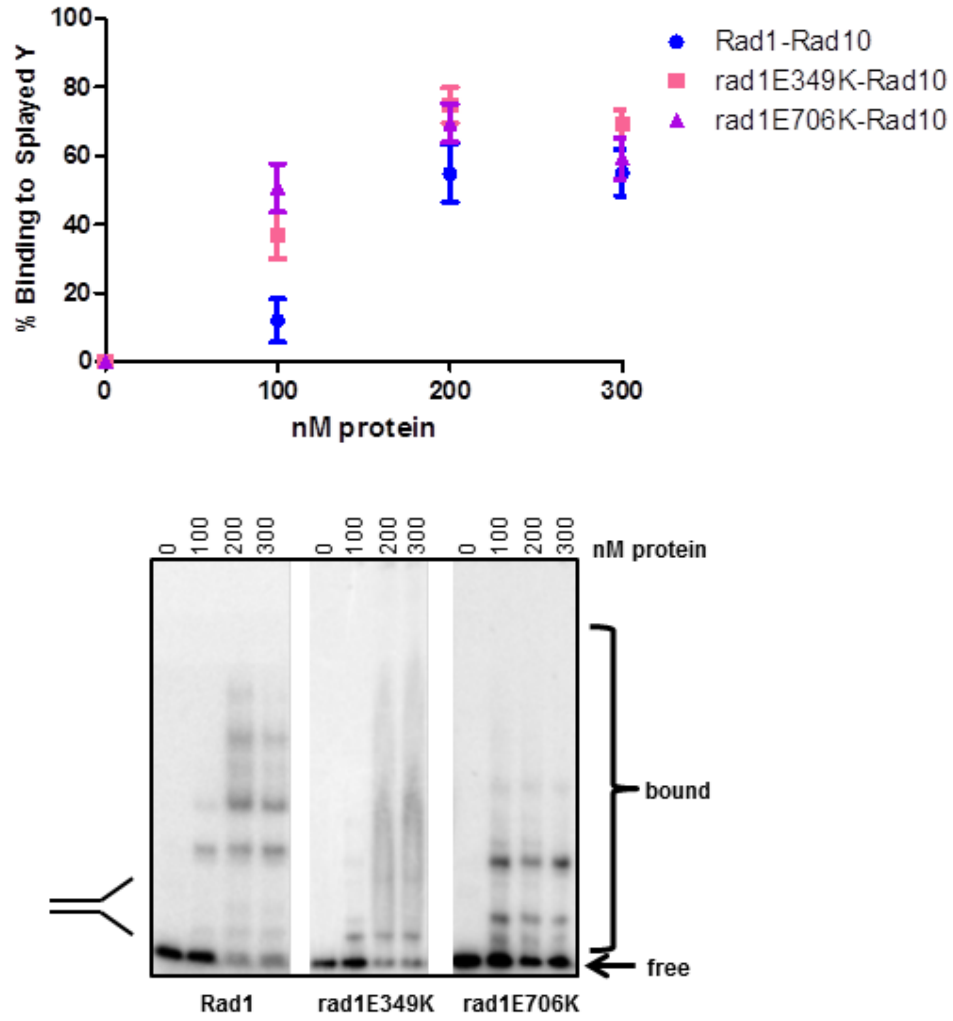

% Binding to Splayed Y
nM protein
Rad1-Rad10
rad1E349K-Rad10
rad1E706K-Rad10
0
100
200
300
nM protein
bound
free
Rad1
rad1E349K
rad1E706K

B

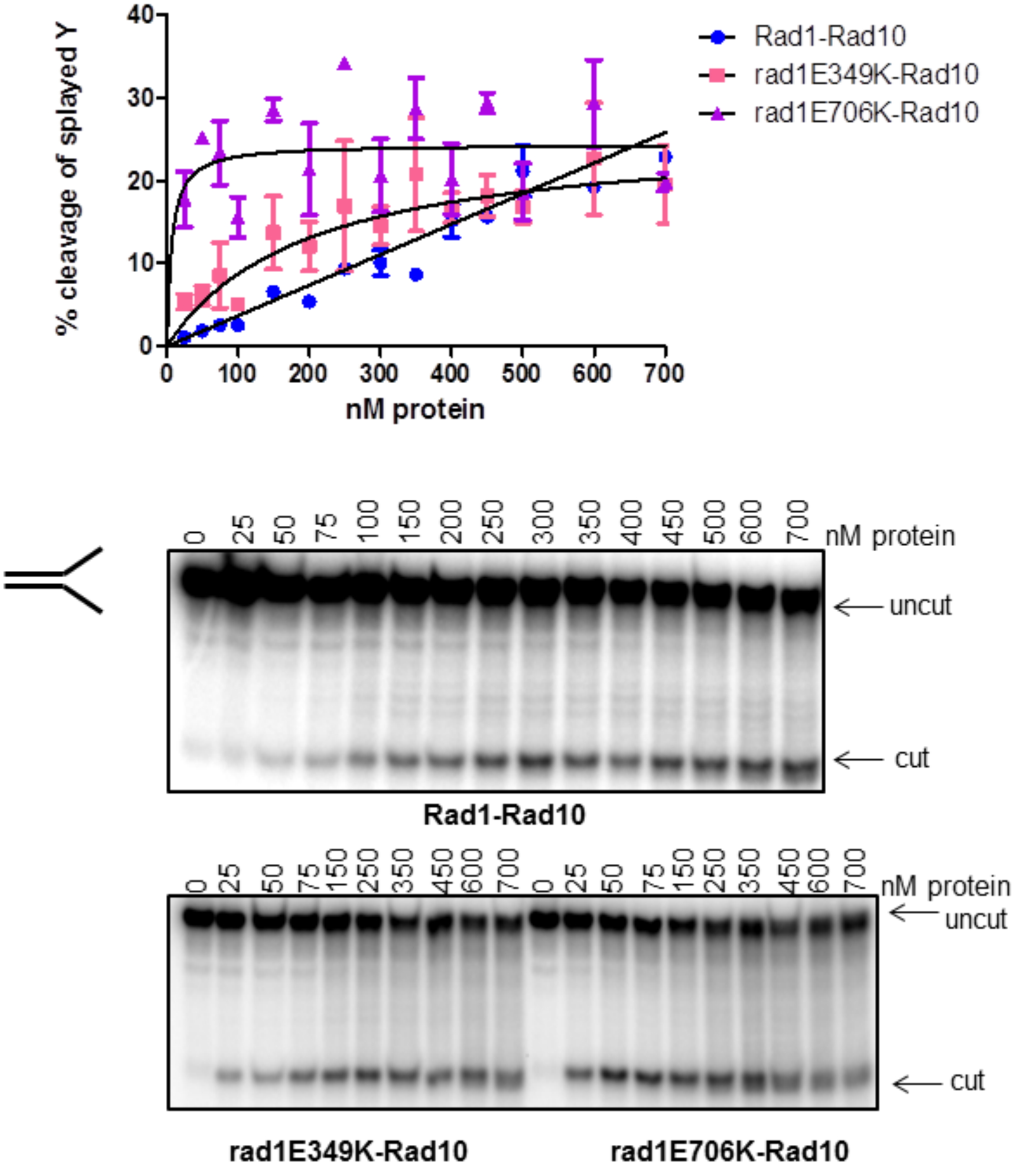

% cleavage of splayed Y
nM protein
Rad1-Rad10
rad1E349K-Rad10
rad1E706K-Rad10
0 25 50 75 100 150 200 250 300 350 400 450 500 600 700 nM protein
uncut
cut
Rad1-Rad10
0 25 50 75 150 250 350 450 600 700 0 25 50 75 150 250 350 450 600 700 nM protein
uncut
cut
rad1E349K-Rad10
rad1E706K-Rad10

C

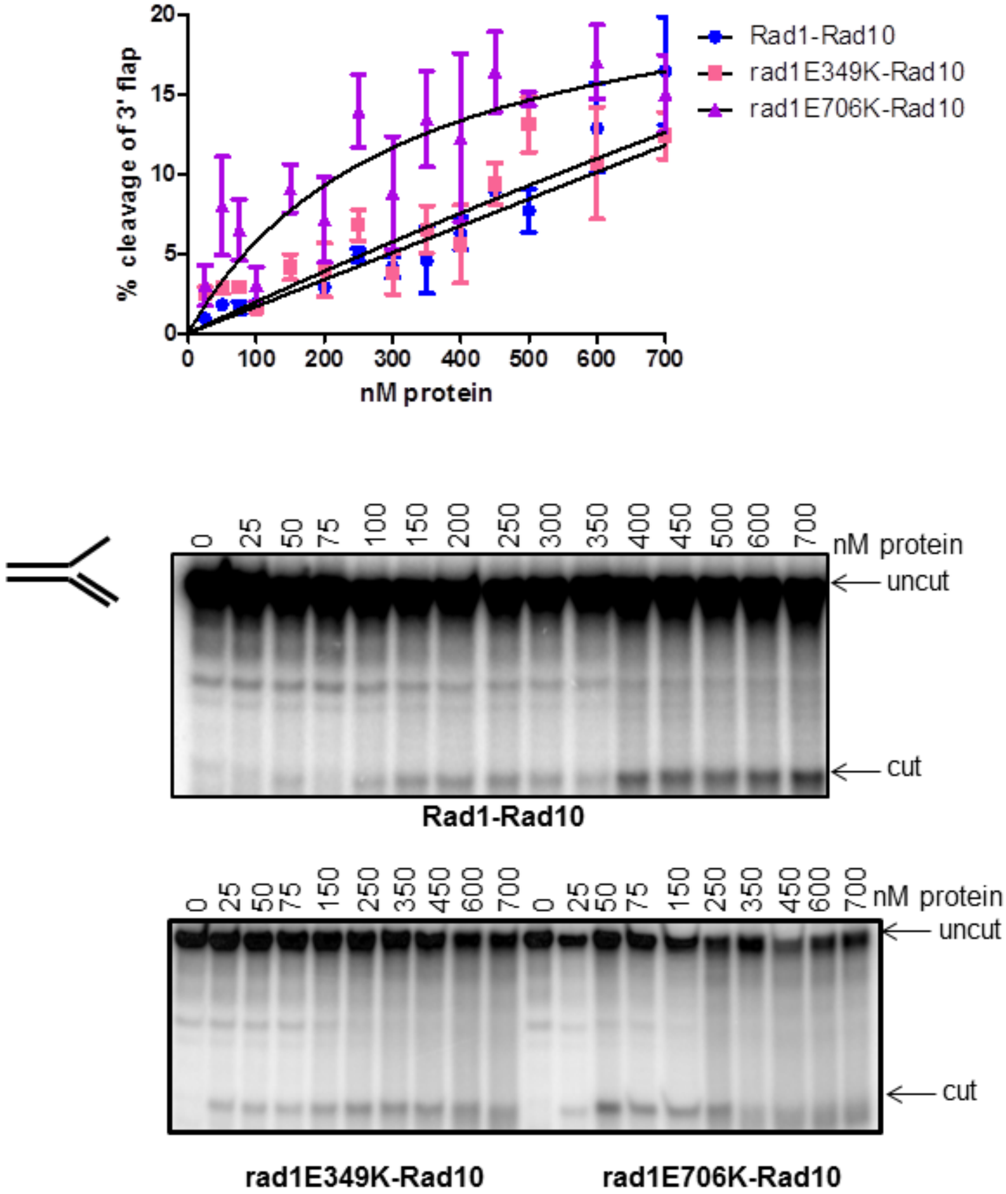

% cleavage of 3' flap
nM protein
Rad1-Rad10
rad1E349K-Rad10
rad1E706K-Rad10
0 25 50 75 100 150 200 250 300 350 400 450 500 600 700 nM protein
uncut
cut
Rad1-Rad10
0 25 50 75 150 250 350 450 600 700 0 25 50 75 150 250 350 450 600 700 nM protein
uncut
cut
rad1E349K-Rad10
rad1E706K-Rad10

Supplementary Figure 8 DNA-binding and endonuclease activities of His-rad1E349K/Rad10 and His-rad1E706K/Rad10 are intact. **a** Gel mobility shift assays of His-Rad1/Rad10, His-rad1E349K/Rad10 and His-rad1E706K/Rad10 were performed with splayed Y substrates. While the overall binding affinities are similar to His-Rad1/Rad10, the binding patterns are more variable. Quantification of percent binding activity in gel mobility shift assays is shown in the upper panel. Representative gels are shown below. **b** Endonuclease activity was determined by endonuclease assays with His-Rad1/Rad10, His-rad1E349K/Rad10 and His-rad1E706K/Rad10 purified from *E. coli*. The splayed DNA substrate (**b**) or the 3' flap DNA substrates (**c**) was incubated with increasing concentrations (25-700 nM) of protein. Percent endonuclease activity was determined. Representative endonuclease assays are shown on the right of each panel. Both mutant complexes retain endonuclease activity. His-rad1E706K/Rad10 appears to have higher cleavage activity that His-Rad1/Rad10, although the cleavage activity is variable. For each set, quantification was performed using ImageQuant 5.2. Data represents the mean ± SEM of at least four independent experiments with multiple protein preparations. His-Rad1/Rad10 (blue), His-rad1E349K-Rad10 (coral) and His-rad1E706K-Rad10 (magenta). Curves shown in (**b**) and (**c**) were based on Michaelis-Menten equations in Prism 5.

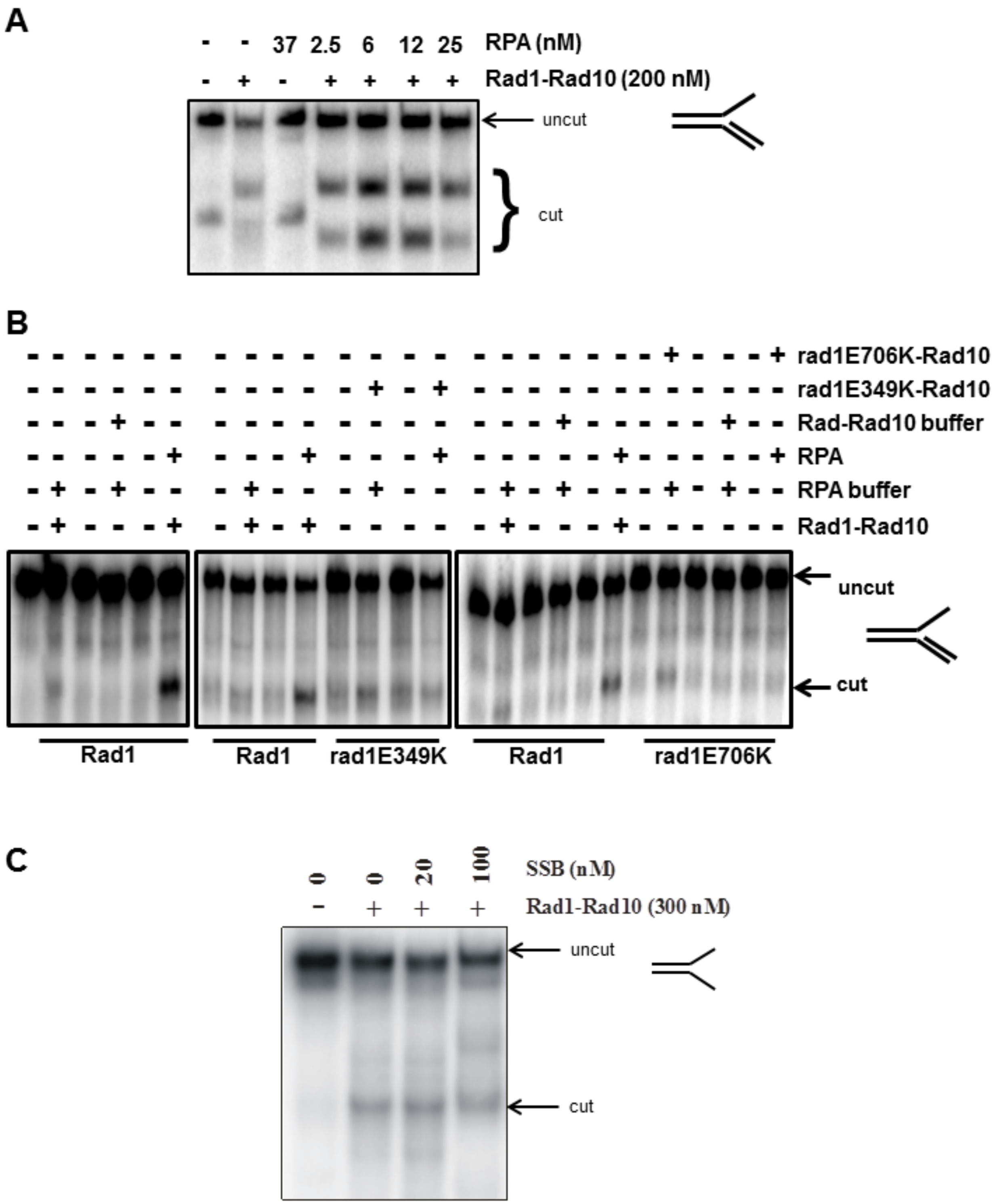
A
- - 37 2.5 6 12 25 RPA (nM)
- + - + + + + Rad1-Rad10 (200 nM)
uncut
cut
B
rad1E706K-Rad10
rad1E349K-Rad10
Rad-Rad10 buffer
RPA
RPA buffer
Rad1-Rad10
uncut
cut
Rad1
Rad1
rad1E349K
Rad1
rad1E706K
C
0 0 20 100 SSB (nM)
- + + + Rad1-Rad10 (300 nM)
uncut
cut

Supplementary Figure 9 **a** Titration of RPA into endonuclease assay of His-Rad1/Rad10. Increasing RPA concentrations stimulate endonuclease activity. **b** Representative gels showing the endonuclease activity of His-Rad1/Rad10, His-rad1E349K-Rad10 and His-rad1E706K-Rad10 (200 nM) in the absence or presence of purified yeast RPA (80 nM). Buffer only controls demonstrate that the buffer alone is not sufficient to cleave the DNA. Similarly, RPA alone has no endonuclease activity. **c** Representative gel of His-Rad1/Rad10 cleavage activity in the absence or presence of *E. coli* SSB. SSB does not stimulate His-Rad1/Rad10 endonuclease activity. The uncut substrate and the cleaved (cut) product are indicated by the arrows to the right of the gel. The DNA substrate used in each panel is indicated to the right of each gel.

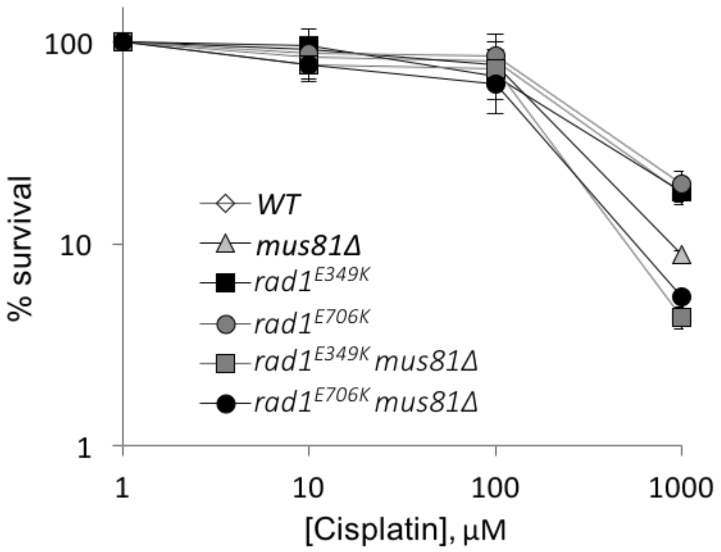

Supplementary Figure 10 CDDP sensitivity of cells expressing rad1-E349K and rad1-E706K. Cells with indicated genotypes were grown to mid-log phase and treated with indicated concentrations of CDDP. Percentage of survival is calculated by dividing the number of colonies growing on CDDP containing media by the number of colonies on YEPD plate without CDDP. The results are the average ± s.d. of three independent experiments.

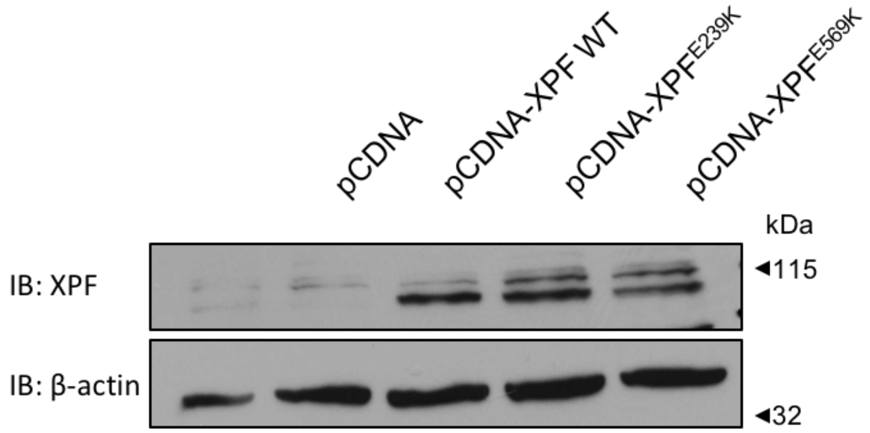

Supplementary Figure 11 The level of expression of FA causing XPF mutants in UV41 cells. XPF and the mutants were detected by immunoblot of whole cell lysates using anti-XPF antibody (Bethyl Laboratories).

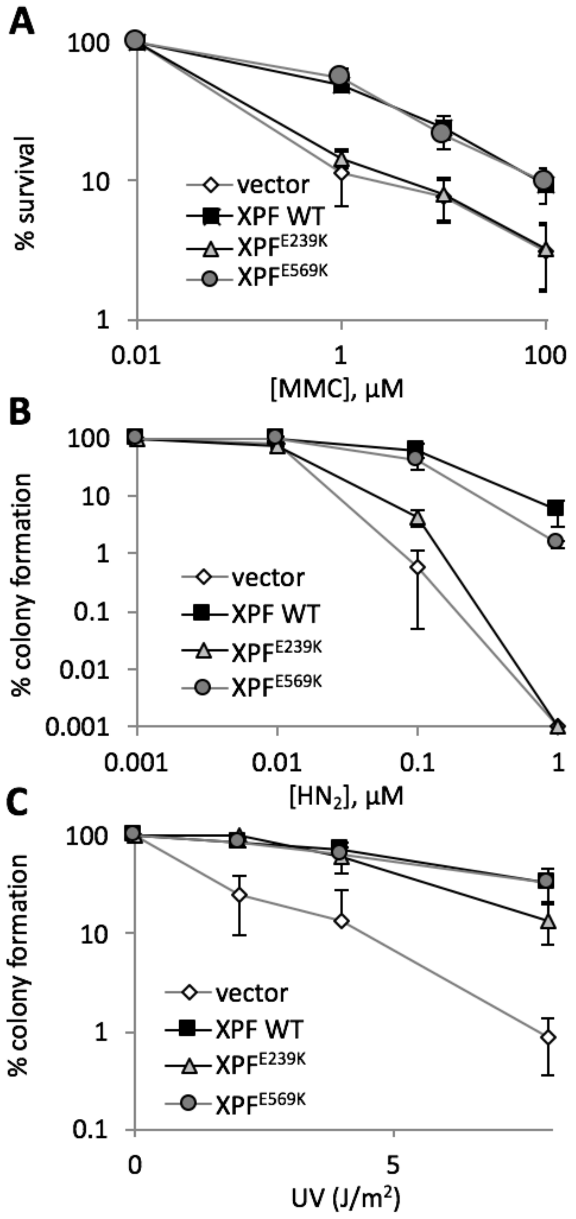

Supplementary Figure 12 a The percentage of survival of XPF^{E239K} and XPF^{E569K} mutants to different doses of Mitomycin C treatment. **b-c** The results of clonogenic assay showing cell survival of XPF^{E239K} and XPF^{E569K} mutants to different doses of HN_2 (**b**) or UV (**c**) treatment. The results are the average $\pm$ s.d. of three independent experiments.

Fig 3d

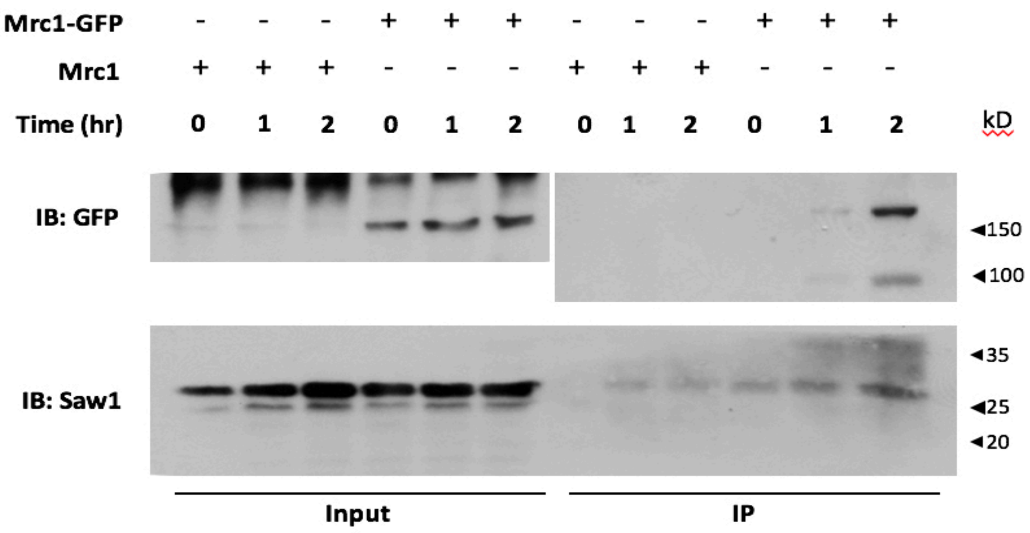

Fig 4d

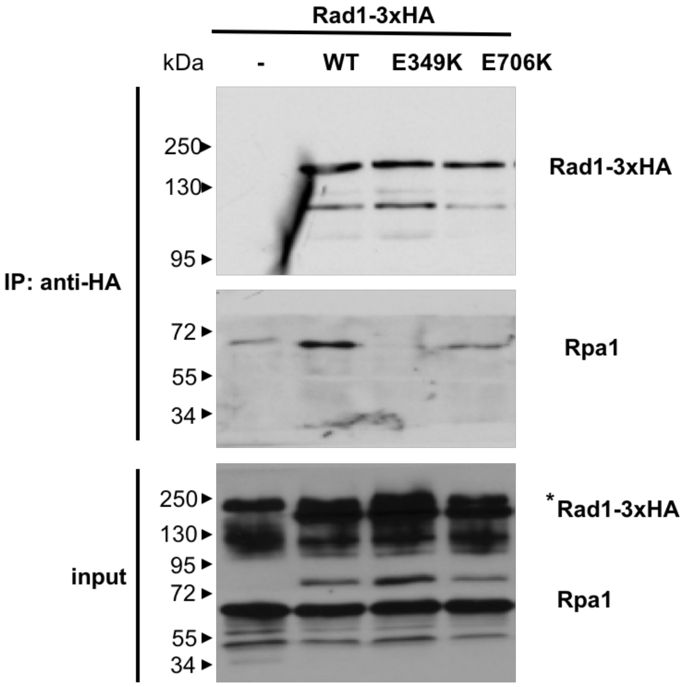

Supplementary Figure 13. Raw data of blots for Fig. 3d and 4d

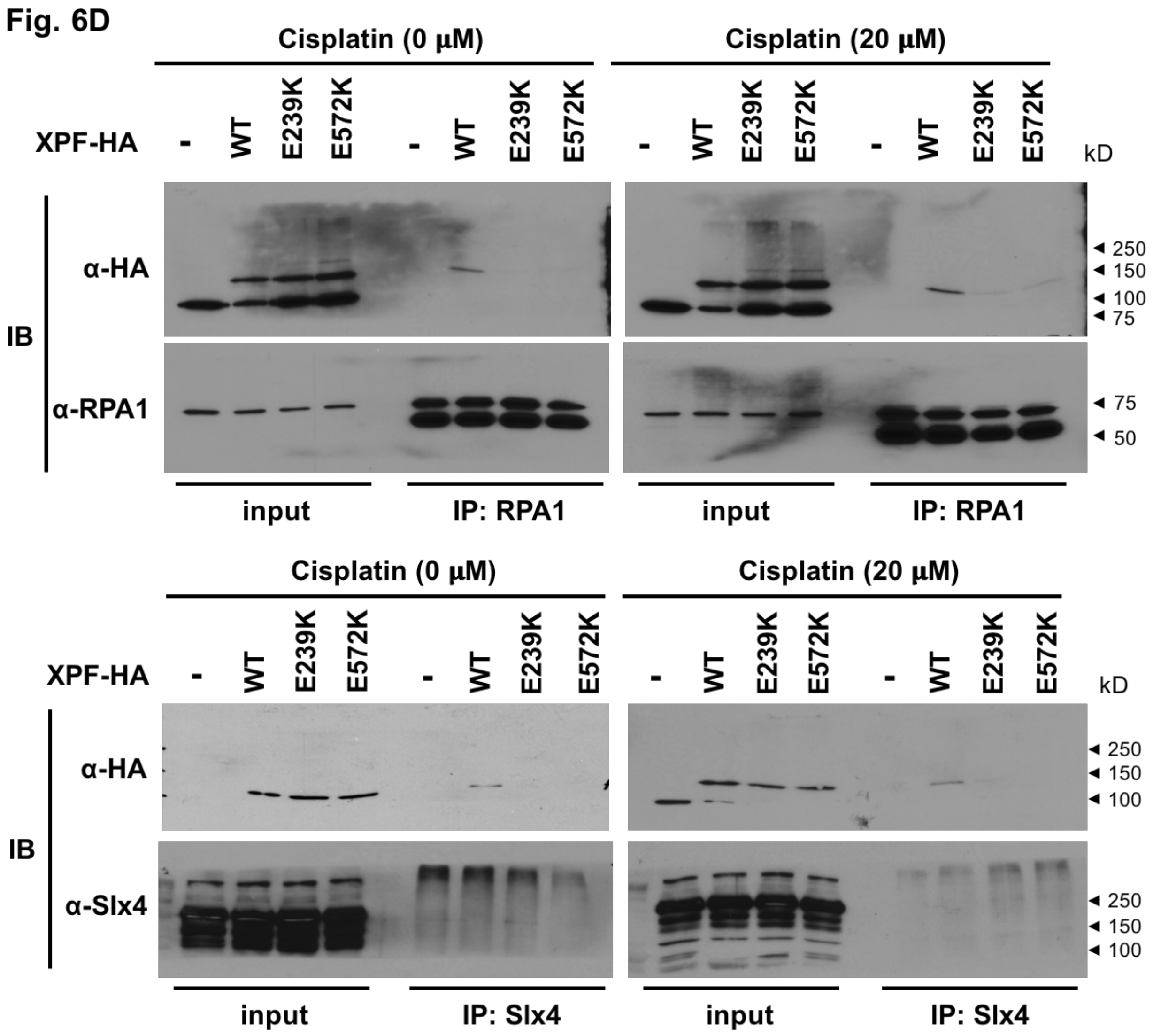

Supplementary Figure 14. Raw data of blots for Fig. 6d

Supplementary Figure 8a EMSAs with splayed substrate

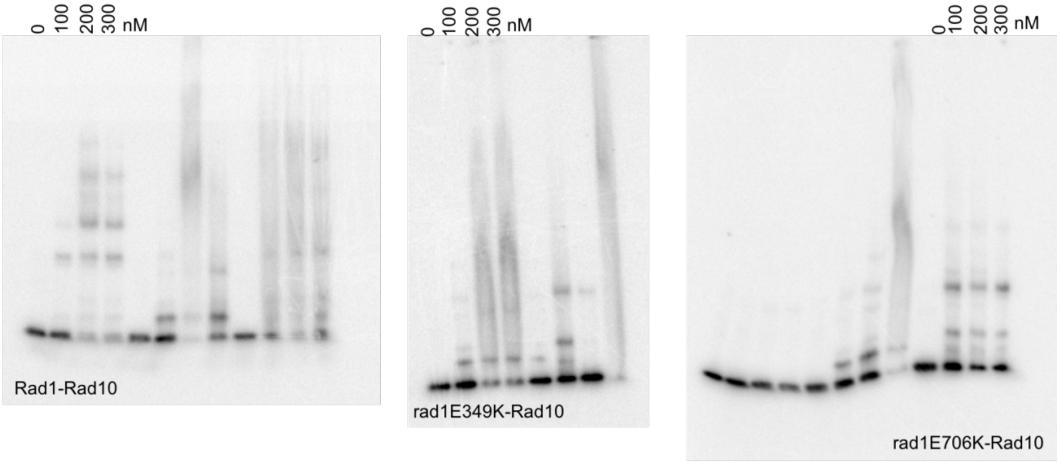

Supplementary Figure 8b Endonuclease assays with splayed substrate

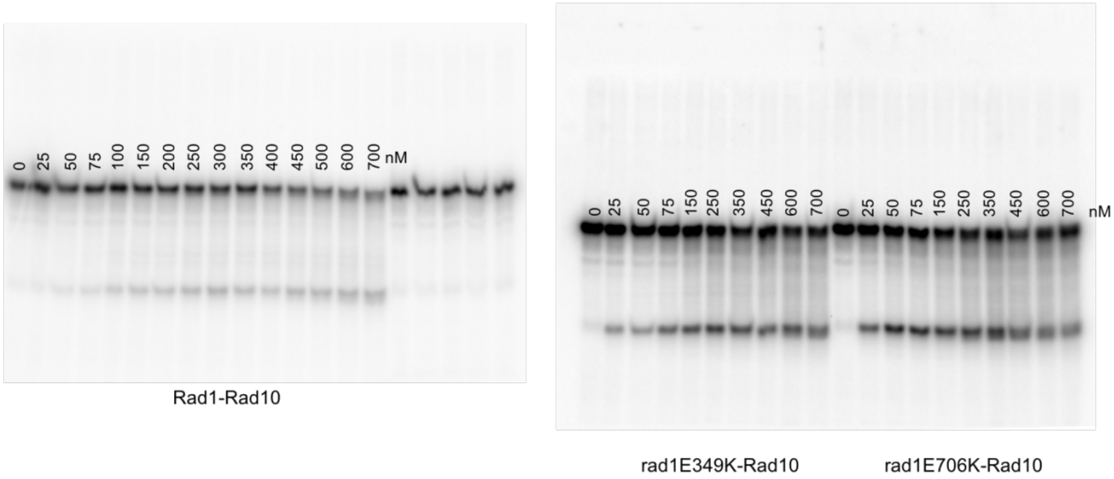

Supplementary Figure 8c Endonuclease assays with 3' flap substrate

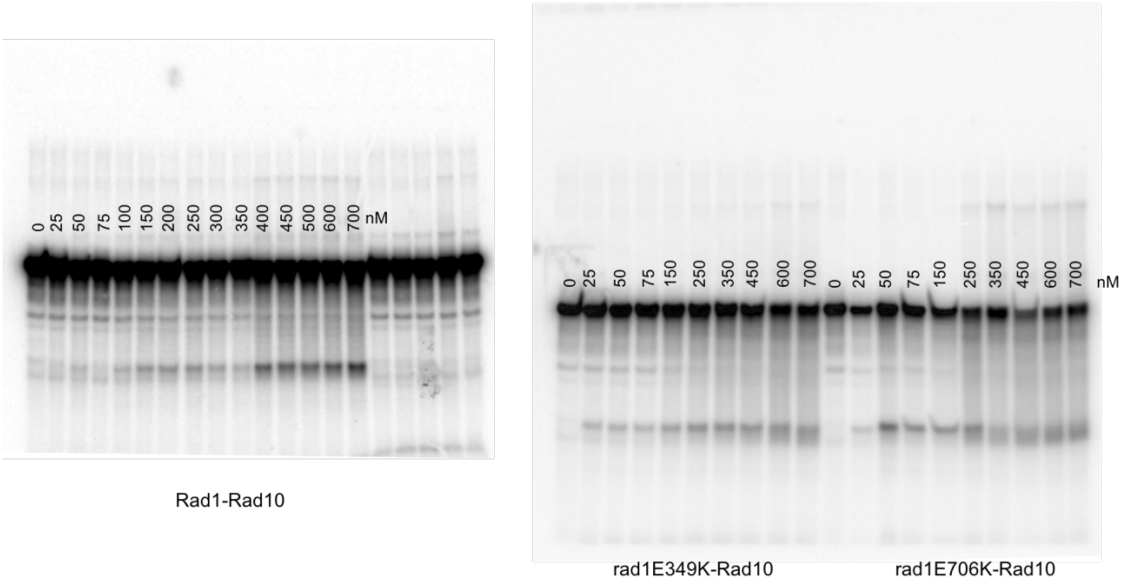

Supplementary Figure 15. Whole gel images for supplementary Figure 8

Supplementary Figure S9a Endonuclease assays with and without RPA

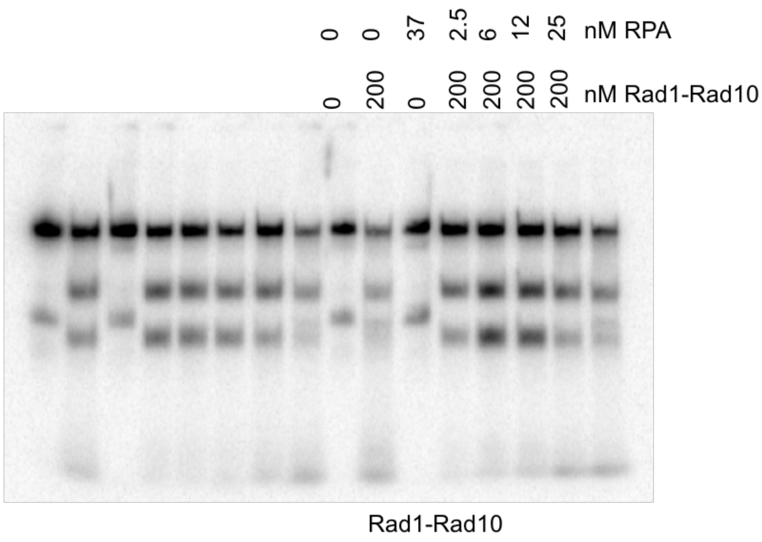

Supplementary Figure 9c

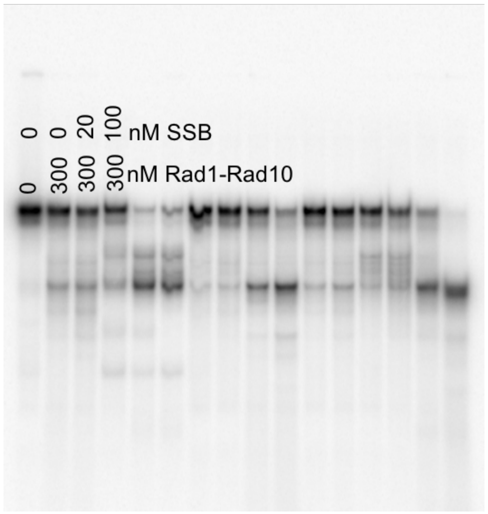

Supplementary Figure 9b

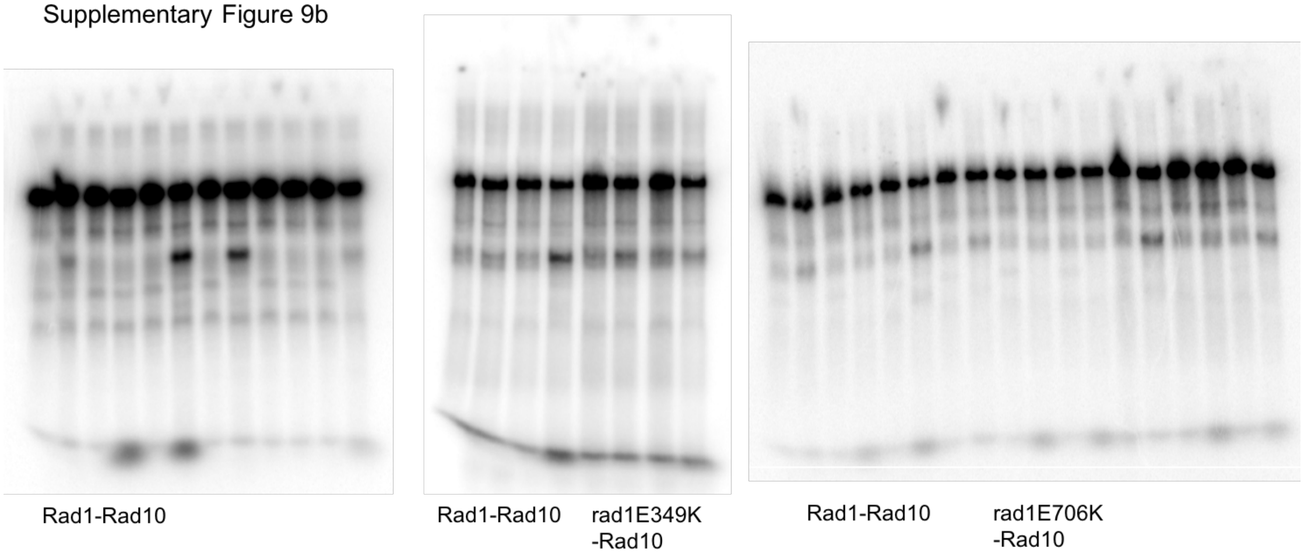

Supplementary Figure 16. Whole gel images for Supplementary Figure 9

Supplementary Table 1. Genotypes of yeast strains used in this study

Strain name	Genotype	Reference
BY4741	MATa, *his3Δ1, leu2Δ0, met15Δ0, ura3Δ0*	[1]
SLY4906	BY4741 *saw1Δ::SAW1-GFP-HIS3*	This study
SLY5676	BY4741 *saw1Δ::SAW1-GFP-HIS3, RAD52-mRFP::NAT*	This study
SLY4932	BY4741 *saw1Δ::NAT*	This study
SLY5616	BY4741 *saw1Δ::SAW1-GFP-HIS3, rad14Δ::KAN*	This study
SLY6020	BY4741 *saw1Δ::SAW1-GFP-HIS3, exo1Δ::KAN*	This study
SLY5838	BY4741 *saw1Δ::SAW1-GFP-HIS3, srs2Δ::KAN*	This study
SLY6034	BY4741 *saw1Δ::SAW1-GFP-HIS3, rad2Δ::KAN*	This study
SLY5613	BY4741 *saw1Δ::SAW1-GFP-HIS3, mph1Δ::KAN*	This study
SLY5622	BY4741 *saw1Δ::SAW1-GFP-HIS3, mus81Δ::KAN*	This study
SLY6111	BY4741 *saw1Δ::SAW1-GFP-HIS3, rad5Δ::KAN*	This study
SLY5619	BY4741 *saw1Δ::SAW1-GFP-HIS3, rad52Δ::URA3*	This study
SLY5839	BY4741 *saw1Δ::SAW1-GFP-HIS3, sgs1Δ::KAN*	This study
SLY5601	BY4741 *saw1Δ::SAW1-GFP-HIS3, pso2Δ::KAN*	This study
SLY5175	BY4741 *mus81Δ::KAN*	This study
SLY5412	BY4741 *mus81Δ::KAN, saw1Δ::NAT*	This study
SLY5822	BY4741 *rad52Δ::URA3*	This study
EAY1141	*hoΔ mat::leu2::hisG hmrΔ3 thr4 leu2 trp1 THR4-ura3-A(205bp)-HOcs-URA3-A ade3::GAL10-HO::NatMX*	[2]
SLY2373	EAY1141 *rad1Δ::RAD1-3HA-KAN*	This study
SLY2346	EAY1141 *rad1Δ::KAN*	This study
SLY2347	EAY1141 *rad1Δ::rad1-E349K-3HA-HYG*	This study
SLY2350	EAY1141 *rad1Δ::rad1-E706K-3HA-HYG*	This study
SLY2372	EAY1141 *rad1Δ::rad1-D825A-3HA-HYG*	This study
SLY2380	EAY1141 *rad1Δ::rad1-D825A, E394K-3HA-HYG*	This study
SLY2381	EAY1141 *rad1Δ::rad1-D825A, E706K-3HA-HYG*	This study
SLY4253	BY4741 *rad1Δ::rad1-E349K-3HA-KAN*	This study
SLY4255	BY4741 *rad1Δ::rad1-E706K-3HA-KAN*	This study
SLY5441	BY4741 *rad1Δ::rad1-E349K-3HA-HYG, mus81Δ::KAN*	This study
SLY5442	BY4741 *rad1Δ::rad1-E706K-3HA-HYG, mus81Δ::KAN*	This study
SLY2301	BY4741 *apn1Δ::KAN*	This study
SLY4235	BY4741 *rad1Δ::URA3*	This study
SLY2307	BY4741 *apn1Δ::KAN, rad1Δ::URA3*	This study
SLY6150	BY4741 *saw1Δ::saw1-D15-GFP-HIS3*	This study
SLY6166	BY4741 *saw1Δ::SAW1-GFP-HIS,3 mrc1Δ::MRC1-mRFP-NAT*	This study
SLY6171	BY4741 *mrc1Δ::MRC1-GFP-HIS3*	This study
AH109	*MATa, trp1-901, leu2-3, 112, ura3-52, his3-200, gal4Δ, gal80Δ, LYS2::GAL1UAS-GAL1TATA-HIS3, GAL2UAS-GAL2TATA-ADE2, URA3::MEL1UAS-MEL1TATA-lacZ*	Clontech

Supplementary Table 1. Countinued

Strain name	Genotype	Reference
Y187	*MATa, ura3-52, his3-200, ade2-101, trp1- 901, leu2-3,112, gal4Δ, met–, gal80Δ, URA3::GAL1UAS-GAL1TATA-lacZ*	Clontech
SLY2374	BY4741 *slx4Δ::SLX4-TAP-HIS3*	This study
SLY5648	EAY1141 *rad1Δ::rad1-C346R-3HA-KAN*	This study
JSY008	BY4741 *saw1Δ::saw1-R19A-KAN*	This study
JSY021	BY4741 *saw1Δ::saw1-R19A-KAN, mus81Δ::HIS3*	This study
JSY059	BY4741 *slx1Δ::KAN*	Invitrogen
JSY023	BY4741 *slx1Δ::NAT, mus81Δ::KAN*	This study
JSY031	BY4741 *slx4Δ::URA3*	This study
JSY033	BY4741 *slx4Δ::URA3, mus81Δ::KAN*	This study
JSY047	BY4741 $slx4^{6A}$ (T72A, T113A, S289A, T319A, S329A, S355A)	This study
JSY048	BY4741 $slx4^{6A}$, *mus81Δ::KAN*	This study
JSY040	BY4741 *saw1Δ::NAT, mus81Δ::KAN, slx4Δ::URA3*	This study
JSY053	BY4741 *slx1Δ::NAT, mus81Δ::KAN, slx4Δ::URA3*	This study
JSY055	BY4741 *slx1Δ::NAT*, $slx4^{6A}$, *mus81Δ::KAN*	This study
JSY057	BY4741 *rad1Δ::rad1-E706K-3HA-HYG, mus81Δ::KAN, slx1Δ::NAT*	This study
JSY058	BY4741 *rad1Δ::rad1-E706K-3HA-HYG, mus81Δ::KAN, slx4Δ::URA3*	This study
JSY043	BY4741 *rad14Δ::HIS3*	This study

Supplementary Table 2. Primers used in this study

Primer name	Sequence (5' to 3')
pNSU2416 F (ChIP)	GCCCAGTATTCTTAACCCAACTGCAC
pNSU2416 R (ChIP)	CAGCTGGCGTAATAGCGAAGAGGCCC
PRE1 F (ChIP)	CCCACAAGTCCTCTGATTTACATTCG
PRE1 R (ChIP)	GGAATTCACCGCATGGTTTTCATAA
SAW1 A	ATGAGTGGAAAAAAGGGCAATGAAAT
SAW1 D	GGCACAGAAACAAAGAGCAGAAGACA
RAD14 A	AGGAAAGTGAAAAGAAGGTAAGGAA
RAD14 D	GGATATCAATCAATGAACACAATCA
EXO1 A	CCAAACTAAGTTCGCGTTTTATTTA
EXO1 D	CTCGCAAAACATATAAAGTTGTCCT
SRS2 A	TTAAAACATGCTAGGGTAACGAGAC
SRS2 D	ACTATTTTTGACTGGGTACTGCTTG
RAD2 A	AATACCACATTTTGTTGCTGTTTTT
RAD2 D	TGTTACTTGTATTGCGATCACTCAT
MPH1 A	CTGGTAAAACAAGATTGCTTTCATT
MPH1 D	TTACTTTTCGGAGTGTAGGAAATTG
MUS81 A	TAATGTATTGGGCACCTTTTATGTC
MUS81 D	TGTTTCAATGACATATCTGAGCACT
RAD5 A	AAATCAAAATGAAGTAAAACCCCTC
RAD5 D	TGGCTGGAAAACTTTCATCTACTAC
RAD52 A	GTACGTGTACCGTGGATTCAACAA
RAD52 D	TTGTTTTTCCGAGTTGCCATATTT
SGS1 A	CCTGATCTAAAAGCTGATATACGGA
SGS1 D	GAAAATTCGAAGTTGATAACTGAGC
PSO2 A	AATTCTGCCAATCTATTTGATCTTG
PSO2 D	CACCAGATAGAGTTTTGAACGAAAT
RAD1 A	CTTTATTTTGCGACTTTTCTTCATC
RAD1 D	TAATGAATATGATTGTGCGCTTCTA
APN1 A	CGAAAAGAGCACAAGAAAATAAAAA
APN1 D	AACTTTACCCTTTTCGTCTGCTAAT
MRC1 A	AGGAAGAGAGAGCAAAAGATTTCAT
MRC1 D	GAAAACGCCAACTAGTGATGTATCT
SLX4 A	GTTCGATGAAAAATCACCAGTAGAG
SLX4 D	TAAAGTAAAGGCTTTGCAGAGAATG

1. Brachmann CB, *et al.* Designer deletion strains derived from Saccharomyces cerevisiae S288C: a useful set of strains and plasmids for PCR-mediated gene disruption and other applications. *Yeast* **14**, 115-132 (1998).

2. Toh GW, *et al.* Mec1/Tel1-dependent phosphorylation of Slx4 stimulates Rad1-Rad10-dependent cleavage of non-homologous DNA tails. *DNA Repair (Amst)* **9**, 718-726 (2010).
